# A Scoping Review of Preventive and Treatment Interventions of Parental Psychological Distress in the NICU in the United States

**DOI:** 10.3390/ijerph22101592

**Published:** 2025-10-20

**Authors:** Kiara A. I. Barnett, Ahnyia Sanders, Rebecca Kyser, Bahar Babagoli, Deepika Goyal, Huynh-Nhu Le

**Affiliations:** 1 Department of Psychological and Brain Sciences, George Washington University, Washington, DC 20006, USA; hnle@gwu.edu; 2 Chan Medical School, University of Massachusetts, Worcester, MA 01655, USA; ahnyia.sanders@umassmed.edu; 3 Himmelfarb Library, George Washington University, Washington, DC 20052, USA; r.kyser@email.gwu.edu; 4 Department of Psychology, Chapman University, Orange, CA 92866, USA; babagoli@chapman.edu; 5 School of Nursing and Health Professions, University of San Francisco, San Francisco, CA 94117, USA; dgoyal@usfca.edu; 6 Santa Clara Valley Medical Center, San José, CA 95128, USA

**Keywords:** NICU, parental distress, psychological interventions, preterm infants, cognitive behavioral therapy, mental health

## Abstract

Parents of premature infants in the Neonatal Intensive Care Unit (NICU) are at elevated risk of anxiety, depression, stress, and trauma, which may impair bonding and infant development. This scoping review synthesized preventive and treatment interventions designed to reduce parental psychological distress in the United States. Guided by PRISMA-ScR, systematic searches were conducted in PubMed, Scopus, MEDLINE, and PsycINFO. Eligible studies were those that examined interventions for parents of preterm infants (<37 weeks’ gestation) initiated before, during, or within one year after NICU discharge. Excluded were studies limited to abstracts or qualitative designs; those not addressing parental depression, anxiety, post-traumatic stress disorder, or stress; and those involving congenital anomalies or conducted outside the United States. Eighteen studies met the inclusion criteria, including ten prevention-focused and seven treatment-focused studies. Eight reported significant reductions in distress, with cognitive behavioral therapy (CBT) and the Creating Opportunities for Parent Empowerment (COPE) program showing the strongest evidence. However, most interventions targeted mothers, highlighting underrepresentation of fathers. Overall, findings underscore the need for interventions that address both parents, include diverse populations, and evaluate participant engagement to improve clinical applicability.

## 1. Introduction

The unexpected birth of a premature infant, defined as birth < 37 weeks’ gestation, that results in admission to the Neonatal Intensive Care Unit (NICU) is a stressful event that places parents at heightened risk of psychological distress (anxiety, depression, stress and trauma) [1,2,3]. Parents of premature infants in the NICU confront numerous challenges, including prolonged separation from their infant, uncertainty about their infant’s health, worry about long-term complications, unfamiliar sights and sounds of the NICU, and parental role alteration [4,5]. Consequently, these parents are at an elevated risk of clinically significant levels of depression, anxiety, and symptoms of posttraumatic stress during their child’s hospital stay. The emotional challenges faced by parents of infants admitted to the NICU can contribute to difficulties with bonding and breastfeeding [6,7]. Consequences of untreated psychological distress extend beyond the NICU, contributing to overprotective and inconsistent parenting behaviors that can hinder parent–child relationships and child development [6,7,8,9,10,11].

Studies on the psychological impact of parents with children in the NICU have largely focused on maternal distress. A recent review of 40 global articles, spanning countries such as Spain, India, and the United States (US), indicated that mothers of preterm infants admitted to the NICU experienced prevalence rates of 31% for symptoms of depression, 51% for anxiety, and 41% for stress [12]. Emerging research highlights that fathers of infants admitted to the NICU also experience psychological distress. Nguyen and colleagues [13], in their systematic review and meta-analysis, found a pooled prevalence of 17.4% for depression and nearly 20% for anxiety among fathers of preterm infants hospitalized in NICUs across diverse countries, including the US, Germany, and Nigeria. Studies also report that 20% to 40% of NICU mothers and fathers of premature and critically ill infants experience trauma-related symptoms [12,14].

### Interventions to Address Parental Psychological Distress

A number of interventions have been developed to reduce and/or prevent psychological distress among NICU mothers [15]. A systematic review and meta-analysis of 12 studies conducted in countries including Brazil, Canada, and the US found that interventions targeting maternal distress had a significant impact on reducing symptoms of depression, with cognitive behavioral therapy (CBT) interventions showing the most promise [15]. Studies covered in this review encompassed a range of approaches, such as psychotherapeutic strategies (i.e., CBT and education-based programs). However, the same interventions did not significantly reduce anxiety symptoms [15].

Although studies report promising evidence for interventions that target psychological distress in the NICU, investigators have largely focused on maternal distress [16,17,18,19]. However, this focus on mothers overlooks fathers who also face psychological distress in the NICU [13] and misses the opportunity to strengthen support systems for mothers. Therefore, it is imperative to identify effective interventions that reduce psychological distress in both mothers and fathers.

To date, a significant amount of research examining interventions for mental health among parents of premature infants in the NICU has been conducted in countries outside the US (e.g., Canada, Israel, United Kingdom) [15,20]. However, US-based healthcare settings, including NICUs, have unique characteristics compared to other developed nations, such as significantly shorter parental leave [21]. Given these considerations, the purpose of this scoping review is to map the literature on interventions that addressed the prevention and treatment of psychological distress among parents (both mothers and fathers) of preterm infants admitted to the NICU in hospitals in the US. In addition, we aimed to identify gaps in the literature in the development, evaluation, and implementation of interventions. The following research question guided our review ‘What interventions have been used to treat or prevent psychological distress in parents of infants in the NICU in the United States?’.

## 2. Materials and Methods

We used the scoping review methodology outlined in Peters et al. [22], which includes the following steps: identifying the review question based on the population, concept, and context; development of inclusion criteria; submission of protocol; identifying relevant studies; study selection; charting the data; and collating, summarizing, and reporting the results. We developed a review protocol to identify research studies that examined interventions that reduce parental distress among parents of preterm infants hospitalized in the NICU in US settings.

### 2.1. Search Strategy

We followed the PRISMA-ScR guidelines for reporting in scoping reviews. The protocol for this systematic review was published in Open Science Framework (OSF) Registries and is available on OSF at https://doi.org/10.17605/OSF.IO/RZKX6 (accessed on 20 April 2025). The search strategies were developed with the assistance of a research librarian. We executed our search strategies in four electronic databases on 1 April 2025 (PubMed, Scopus, MEDLINE (via PubMed), and PsycINFO).

### 2.2. Eligibility Criteria

Peer-reviewed primary research articles that reported on interventions addressing parental depression, anxiety, stress, and/or post-traumatic stress disorder (PTSD) occurring before, during, or up to 1 year after NICU discharge were included. We included studies that reported newborn gestational age or birthweight to avoid limiting the inclusion of potentially relevant populations. Studies were included if they reported on preterm infants (<37 weeks’ gestation), very preterm infants (<32 weeks’ gestation), very-low-birthweight infants (<1500 g), or low-birthweight infants (<2500 g). Infant birthweights were selected to correspond to births at gestational ages of <32 weeks and <37 weeks, respectively, based on estimated fetal weight charts by the World Health Organization [23,24]. We included studies that reported on randomized control trials (RCT), wait-list control, treatment as usual, standard care, attention control, or other interventions as comparison groups. We also included studies conducted between the years 2000 to April 2025 to compare interventions implemented within a contemporary hospital setting (i.e., physical design, reorganization of care) [25]. We excluded studies that were available only as abstracts or used a qualitative approach. We also excluded studies that did not report on parental depression, anxiety, PTSD, or stress. We also excluded studies of infants diagnosed with congenital heart defects and congenital abnormalities and excluded studies that were conducted outside the US.

### 2.3. Outcomes

Our primary outcomes comprised clinical and psychological interventions targeting parental depressive, anxiety-related, stress-related or trauma-related symptoms and/or clinical diagnoses of depression, anxiety, acute stress disorder or post-traumatic stress disorder. Our secondary outcomes included intervention characteristics: intervention description, mode of delivery, frequency, duration, and length, participant engagement, and attrition.

### 2.4. Data Collection and Synthesis

**Figure 1:**
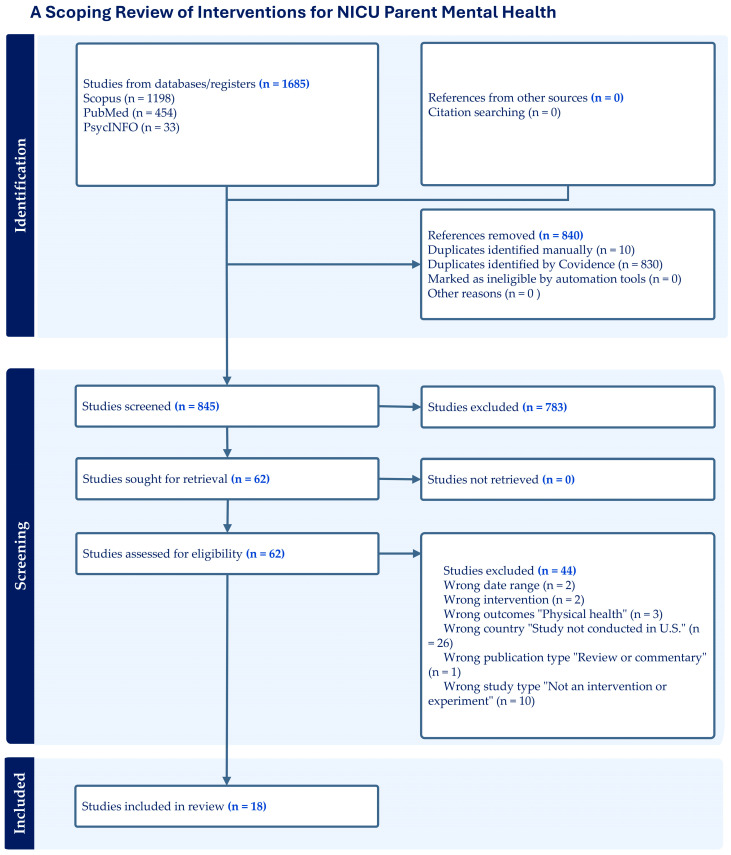
PRISMA-ScR flow diagram.

Data collection and synthesis were completed using Covidence systematic review software (MEL, Australia) [26], a web-based software platform that streamlines the production of systematic and other literature reviews. Two blinded reviewers independently screened titles and abstracts and applied the inclusion and exclusion criteria to the relevant full-text articles. We extracted data on study setting, study design, population baseline characteristics, sample size, measures of psychological distress, type of intervention (e.g., kangaroo care, CBT), intervention delivery method (e.g., in person, e-health), characteristics and outcomes of interventions, and participant engagement (e.g., number of sessions attended). We used the preferred reporting items for systematic reviews and meta-analyses (PRISMA) to guide screening along with the PRISMA extension [27] for scoping reviews checklist [28] (Figure 1). We synthesized and reported data in two categories, intervention characteristics and reported outcomes and participant engagement. Intervention characteristics and outcomes are reported first by prevention interventions and then treatment. Under each category, we classified interventions into four categories: (1) psychotherapeutic; (2) educational–behavioral; (3) educational; and (4) complementary/alternative medicine.

## 3. Results

### 3.1. Study Selection

The search strategy produced 1685 records, of which 845 remained for title and abstract screening after duplicates were removed. Of these, 62 full-text articles were screened using the inclusion and exclusion criteria, and 18 articles that met the inclusion criteria were identified (Figure 1). Intervention type was categorized as prevention, treatment, or not specified. Studies in the prevention category did not require participants to exhibit high levels of symptomatology or meet strict diagnostic criteria according to the Diagnostic and Statistical Manual of Mental Disorders, Fifth Edition (DSM-5) [29]. Studies in the treatment category included a diagnostic interview to identify depressive or anxiety disorders and/or reported the intervention as treatment.

### 3.2. Intervention Characteristics and Outcomes

Table 1 provides a summary of the intervention characteristics of the 18 publications included. Of the 18 studies reviewed, the majority focused on prevention (61.1%), primarily psychotherapeutic (22.2%) and educational–behavioral (27.8%) approaches, while 38.8% were treatment studies, most of which examined complementary or alternative medicine (27.7%). Most studies were conducted in urban settings (83.3%), with only one study spanning both urban and rural contexts and two studies where the setting was unclear. Across all studies, participant race and ethnicity were most reported as White (53.1%) and Black (23.8%), with smaller proportions identifying as Hispanic/Latino (14.3%), Asian (3.8%), multiracial (0.4%), American Indian (0.5%), and Native Hawaiian/Pacific Islander (0.1%).

Table 2 outlines additional details about the characteristics and key findings of the included publications. Most studies (n = 11; 61.11%) employed an RCT design. Eleven (61.1%) articles reported exclusively recruiting mothers [16,17,18,30,31,32,33,34,35,36,37], while none exclusively recruited fathers. A total of four studies (22.22%) involved interventions delivered by graduate students or registered nurses [30,35,38,39]. Five articles (27.78%) reported interventions that were conducted by mental health professionals or graduate students [16,30,35,36,37], and four studies (22.22%) were led by allied health professionals, such as occupational therapists [17,40,41,42]. Additionally, three studies (16.67%) utilized self-guided interventions [31,33,43]. Out of the 18 studies, nine (50%) incorporated various forms of technology, such as mobile apps and audiotapes, to enhance parent outcomes [17,31,32,33,35,36,38,39,43].

### 3.3. Prevention Interventions

Eleven studies (61.11%) investigated the efficacy or effectiveness of interventions aimed to prevent psychological distress [16,17,18,32,33,35,36,37,38,39,43]. Of these eleven articles reporting on preventive interventions, four (27.27%) were categorized as psychotherapeutic, which were based on CBT [16,35,36,37]. Five (27.78%) were categorized as educational–behavioral [17,18,32,38,39] and two as educational [33,43].

#### 3.3.1. Psychotherapeutic

Interventions categorized as psychotherapeutic employ therapeutic modalities such as CBT, typically facilitated by a mental health professional. Among the three prevention interventions, all psychotherapeutic approaches were based on CBT and showed promising mental health outcomes [16,35,36]. However, the results yielded mixed levels of statistical significance. In a sample of 50 mothers, Bernard and colleagues [16] found that an individual CBT intervention, consisting of 10 sessions over two weeks, exhibited a trend towards reduced trauma and depressive symptoms at follow-up, although the difference was not statistically significant. The intervention significantly reduced maternal anxiety levels. However, changes in depression and trauma symptoms were not statistically significant. Thus, while the intervention effectively reduced anxiety, it had limited impact on depression and trauma symptoms.

Shaw et al. [35] investigated the effects of an intervention consisting of six sessions of manualized Trauma-Focused CBT delivered over 3 to 4 weeks, with one or two sessions each week. The results indicated that this intervention significantly reduced symptoms of trauma and depression in mothers of preterm infants. The authors’ subsequent publication in 2014 [36] presented 6-month follow-up outcomes, indicating that the intervention had significant reductions in symptoms of trauma and depression. Regarding anxiety symptoms, while both groups experienced a decline in scores, the intervention group exhibited a more significant reduction than the control group.

#### 3.3.2. Educational–Behavioral Interventions

Educational–behavioral interventions aim to equip parents with the knowledge and skills to engage with their preterm infant or to actively manage psychological distress and directs parents to modify behaviors. Among the six educational–behavioral studies classified as preventive interventions, one study explored the effects of mobile-enhanced Family Integrated Care (mFICare), and one was based on CBT. One compared the effectiveness of auditory–tactile–visual–vestibular stimulation to kangaroo care. Three focused on the Creating Opportunities for Parent Empowerment (COPE) program.

Franck et al. [17] compared the effectiveness of mobile-enhanced Family Integrated Care (mFICare) with traditional family-centered care on preventing symptoms of stress, depression, and trauma. mFICare is an advanced neonatal care model that integrates parents into their preterm infant’s care using mobile technology for support and education, while family-centered care in the NICU involves a collaborative approach that actively includes parents in decision-making and care processes. This intervention was unique in its co-development process, which involved former parents of infants admitted to the NICU. The results indicated no clinically significant differences between the two interventions in reducing PTSD or depression symptoms. However, mFICare was found to be more effective in preventing clinically significant PTSD symptoms in mothers experiencing higher levels of NICU-related stress compared to family-centered care alone.

In a pilot RCT, Silverstein and colleagues [37] evaluated a manualized problem-solving intervention based on CBT, consisting of four weekly individual sessions. These sessions were conducted at a location of the mother’s choice (e.g., hospital, home) and included 50 low-income Black and Latina mothers of preterm infants in an urban NICU. The results suggested that the intervention may effectively reduce depressive symptoms among these mothers. The intervention group experienced fewer episodes of moderately severe depressive symptoms compared to the control group over a six-month follow-up period, although these findings were not statistically significant. Holditch-Davis et al. [18] compared the effects of two interventions designed to prevent psychological distress in 240 mothers of preterm infants in the NICU, utilizing an attention control. The interventions included an auditory–tactile–visual–vestibular intervention and kangaroo care. The auditory–tactile–visual–vestibular intervention involves a multi-sensory approach where mothers provide moderate stroking, eye contact, talking, and rocking to their infants over a 15 min period, starting with auditory stimulation and gradually adding tactile and visual components as the infant becomes alert. Kangaroo Care consists of holding the infant in skin-to-skin contact between the mother’s breasts, promoting warmth and bonding. Participants completed standardized measures of depressive and anxiety symptoms at multiple time points, including Baseline (prior to the intervention), Post-intervention (immediately after the intervention), and Follow-up (at 2, 6, and 12 months postpartum, adjusted for the infant’s age). The findings indicated that while neither intervention significantly reduced maternal distress compared to the control group, mothers who engaged in any form of massage, including auditory–tactile–visual–vestibular, experienced a more rapid decline in depressive symptoms.

Notably, the Creating Opportunities for Parent Empowerment (COPE) program demonstrated the most effective outcomes among the preventive interventions [32,39]. COPE is a four-phase program delivered through audiotapes and written materials, designed to provide parents with both information and activities to enhance their knowledge and beliefs about their preterm infants and their own parenting role. The program, which includes educational sessions aimed at increasing parental knowledge and confidence in caring for premature infants, showed significant reductions in depressive and anxiety symptoms (*p*-value not specified). Over half of the participants experienced clinically meaningful improvements and reported high satisfaction with the intervention [32,38,39]. Additionally, COPE was particularly effective for younger mothers, significantly predicting lower levels of anxiety, and for mothers with a history of trauma [32].

#### 3.3.3. Educational

Educational interventions provide parents with information to enhance understanding of birth and preterm infants but do not prompt behavior activation to target distress. Rau and colleagues [33] investigated the effectiveness of providing participants with either a multimedia tablet or a paper handout at their bedside to receive supplemental information during clinical counseling. Results demonstrated that both interventions were equally effective at decreasing depressive symptoms, although parents were less likely to review all the material on the tablet compared to the paper handouts [13].

Erdei et al. [43] conducted a pretest–posttest pilot study to assess the impact of the ‘My Brigham Baby’ educational smartphone application on the psychosocial experiences of parents in a NICU. The app provides educational resources, access to medical records, and tools for logging parenting activities, with participants required to use it at least once per week. The study surveyed a total of 50 participants, with 25 parents before and 25 different parents after the app rollout. Results showed a slight increase in stress scores and a slight decrease in anxiety scores, but neither change was statistically significant.

### 3.4. Treatment Interventions

Seven (38.89%) researchers investigated interventions aimed at treating symptoms of parental psychological distress in the NICU. Two (11.11%) of these studies evaluated psychotherapeutic interventions [30,34]. Five studies (27.78%) explored complementary/alternative medicine modalities to alleviate symptoms of psychological distress [31,40,41,42,44].

#### 3.4.1. Psychotherapeutic

Two psychotherapeutic interventions yielded mixed results [30,44]. Horwitz et al. [30] conducted an RCT to evaluate the effectiveness of a Trauma-Focused CBT intervention, which consisted of either six or nine manualized sessions, with two additional sessions adapted from the COPE program. The intervention aimed to reduce maternal symptoms of anxiety, depression, and trauma, and included education about perceptions of vulnerability among 105 mothers compared to NICU standard care. However, the differences between the intervention and comparison groups were not statistically significant. The study noted that the intervention was particularly effective for mothers with a prior trauma history.

The second psychotherapeutic intervention involved non-directive listening sessions, focusing on empathetic listening and problem-solving, and was evaluated using a pretest–posttest design [44]. This nurse-delivered program consisted of up to six sessions aimed at reducing depressive and anxiety symptoms in mothers of preterm infants hospitalized in a NICU. Results showed clinically significant reductions in depressive and anxiety symptoms in mothers of preterm infants hospitalized in the NICU.

#### 3.4.2. Complementary/Alternative Medicine

We referenced Sabnis et al. [20] who used the National Center for Complementary and Integrative Health’s classifications to identify mind–body practices outside the conventional medical framework. These interventions often include activities like journaling, relaxation techniques, music therapy, infant massage, and acupuncture. Overall, complementary and alternative interventions showed trends toward reducing psychological distress, although small sample sizes (ranging from 20 to 260) may have contributed to non-significant changes [31,40,41,42,44].

Marshall et al. [40] aimed to treat symptoms of stress by conducting a single individual session that taught mindfulness techniques to 28 parents, encouraging them to practice these techniques both at their infant’s bedside and away from the hospital. Although parents’ responses to a seven-item multiple-choice questionnaire indicated that nearly 80% found the mindfulness practice helpful, there were no statistically significant differences in parental stress levels between the pre- and post-mindfulness-based training.

In another study, a self-guided journaling intervention was implemented [44]. The RCT included a sample of 97 parents who were provided journals containing both blank pages and guided prompts to express their thoughts and emotions, with the aim of reducing symptoms of anxiety and depression. These parents were compared to a control group receiving standard care. Parents in the intervention group exhibited a decrease in anxiety, although the reduction was not statistically significant, while depression levels remained unchanged. However, fathers who participated in the journaling program experienced a significant decrease in anxiety compared to mothers.

A mixed-methods study aimed to investigate whether an art-based group utilizing scrapbooking in the NICU could reduce parent anxiety symptoms and to explore the lived experiences of parents participating in the group [41]. The study involved 40 parents, and results indicated a clinically significant decline in state anxiety. Another complementary study was a single-sample, pre–post-test feasibility study aimed at evaluating the effectiveness of a relaxation guided imagery (RGI) intervention in reducing maternal stress [31]. The study involved twenty mothers and assessed their perceived stress, anxiety, and depression symptoms, after an 8-week RGI intervention. The results indicated that the average use of RGI was associated with lower levels of distress, although this correlation was not statistically significant.

Petteys et al. [42] conducted a pilot study involving 55 parent–infant dyads to examine the impact of parent education and participation in mindfulness-based neurodevelopmental care on parent outcomes on stress. Results measuring psychological distress indicated that the experimental group experienced a significant reduction in stress scores compared to a control group receiving standard NICU care.

### 3.5. Participant Engagement

Only four of the 18 studies (22.22%) reported data on participant engagement and intervention completion [31,34,37,40]. Howland et al. [31] reported that their sample of 20 mothers, predominantly White (60%), who were instructed to listen to recordings of guided relaxation at least once daily over the course of 8 weeks, were highly engaged (M = 4.46 daily; SD = 1.1). In a mindfulness training intervention designed to be completed within the first two weeks of the infant’s hospitalization, researchers reported high engagement with various relaxation techniques: Relaxing sighs (M = 4.6; SD = 2.2), Meditation (M = 3.7; SD = 4.1), Breath meditation (M = 2.1; SD = 2.3), Body scan meditation (M = 1.9; SD = 2.1), and Calming phrases (M = 4.2; SD = 2.4). Segre et al. [34] reported that the mean number of listening sessions attended by participants in their six-session group was 5.22 (SD = 1). However, Marshall and colleagues [40] also indicated recruitment challenges, reporting parent perceptions of mindfulness and staff availability as barriers.

Some studies discussed participant engagement, although data were not reported. Holditch-Davis et al. [18] reported that although participants engaged in the intervention, many mothers withdrew from the study by hospital discharge. In a self-guided journal intervention, 76% of participants reported journaling at least a few times throughout the study [44]. In an art-based intervention, 88.4% completed the 2 h scrapbooking groups that were facilitated over five months [41]. Shaw et al. [35,36] indicated that more than 90% of mothers completed all six sessions of a manualized Trauma-focused CBT program. Although one study examining the COPE program reported high retention, during recruitment, a significant portion of eligible families refused participation, with most parents citing reasons such as being too stressed or tired to participate, wanting to concentrate only on the new infant, or believing that participation would take time away from other children at home [38].

## 4. Discussion

This scoping review of interventions targeting psychological distress among parents of preterm infants in the NICU in the US yielded 18 studies that report on the efficacy or effectiveness of interventions reducing symptoms of stress, anxiety, depression, and trauma. Eleven studies were classified as prevention interventions, and seven were categorized as treatment-based interventions. Of the 18 studies, eight studies report statistically significant reductions of stress, anxiety, depression, and/or trauma symptoms [30,32,33,38,39,44]. Our findings confirm previous research indicating that CBT interventions and the COPE program were most effective at reducing symptoms of psychological distress among mothers [15,20]. However, all studies examining CBT and COPE included only mothers.

Although nearly half of preventive and treatment interventions did not find statistically significant effects on measures of psychological distress, results generally demonstrated positive trends towards reducing symptoms of stress, anxiety, depression, and trauma. It is likely that these results are indicative of small sample sizes across studies, warranting replication of various interventions with larger sample sizes. Behavioral based interventions, in particular journaling, mindfulness, and relaxation techniques, yielded promising qualitative results. Parents participating in these studies reported it was a beneficial way to express difficult emotions, helped them to focus on themselves, and reduced their anxiety [31,40,41,44]. In particular, the journaling intervention was found to reduce anxious symptoms significantly more for fathers than mothers [44].

Despite increasing research on paternal distress in the NICU [12,13,14], our review found that only seven of the 18 articles included fathers. Our findings are consistent with current literature, emphasizing that intervention development and implementation continue to lag in the inclusion of fathers [13,20]. This is concerning as fathers of premature infants face unique stressors. They are often the first parent to encounter the stressful NICU environment as they accompany their infant during admission while their birthing partner recovers [45,46,47,48]. Fathers must also balance concern for their infant’s health and support for their birthing partner [45,48,49]. Fathers tend to minimize their outward emotional response, instead directing their energy to supporting their partner [50,51,52,53]. Their role as a caregiver and support person may overshadow their emotional needs and leave little room to explore those emotions.

Results from this scoping review showed that brief, low-demand interventions like mindfulness, COPE, journaling, and listening sessions had high participant engagement. These interventions require short, accessible lessons with minimal interaction with trained staff and encourage parents to engage at the infant’s bedside. However, high engagement was accompanied by recruitment challenges and attrition due to elevated stress, the need to be present at the infant’s bedside, managing other responsibilities, and work obligations [32,38]. The use of technology may address these distinct barriers to service. Given that nearly half of the interventions in our review utilized technology (e.g., apps, audiotapes) and these interventions showed promising results, further investigation into how technology can be used to overcome service barriers in this population is warranted.

Our results also indicate that while racial/ethnic minorities were included in nearly all of the studies, White parents were most represented. Among the 13 studies that provided comprehensive race/ethnicity data for all participants, White parents, predominantly mothers, were represented across all studies (53.1%). Although some study samples may reflect the racial and ethnic demographics of the US general population, they do not accurately represent NICU admissions across different racial/ethnic groups [54,55,56]. Reproductive health disparities are evident in the NICU [56], with preterm birth rates being highest among Black and Native American populations in the US, at 14.6% and 12.2%, respectively [57]. These findings underscore the importance of making special efforts to recruit samples that reflect the current NICU census.

Our review revealed that most studies were conducted in NICUs that are located in urban environments. Families residing at a considerable distance from the NICU face additional challenges in visiting and participating in interventions such as family-centered care [58,59]. This observation provides novel insights into the geographical distribution of existing studies, highlighting a lack of diversity with most studies concentrated on the West Coast or in the Midwest. Given the predominance of urban settings in these studies, it is crucial to consider parents who must travel long distances from rural communities or hospital deserts to reach the NICU.

## 5. Strengths and Limitations

Our scoping review has several strengths and limitations. Regarding strengths, we conducted an extensive literature search of relevant studies. Additionally, we included fathers, who are often overlooked in the perinatal period, particularly in the NICU where the focus is primarily on the needs of the preterm infant and the emotional challenges faced by mothers. Regarding limitations, our study does not account for the sociocultural differences across communities, as it is restricted to studies conducted in a single country, potentially overlooking how cultural practices influence family support and adaptation for parents with a preterm newborn. The limited reporting on participant engagement, such as session completion, is a notable finding that highlights a gap in the literature. Additionally, nearly half of the studies did not report significant findings, which presents challenges in assessing the most effective and feasible interventions. Although fathers were included in our review, the majority of the studies focused solely on mothers, limiting generalizability of the findings to fathers of preterm infants in the NICU. While our review did not exclude studies based on family structure, all parent-dyad studies identified focused exclusively on heterosexual couples.

## 6. Implications for Future Studies

Findings from this scoping review has several implications for future studies. First, future studies should replicate existing research with larger samples that not only reflect the current racial/ethnic demographics of the NICU population but also include recruitment strategies to enhance the representation diverse family compositions and fathers. Second, interventions aimed at engaging fathers should incorporate self-guided and expressive strategies, such as journaling, as these opportunities for emotional processing may be particularly beneficial. Third, future studies should incorporate data on parents’ geographical location and proximity to the NICU to enhance the context of study findings and develop interventions that better serve parents facing geographical disadvantages. Fourth, intervention development should continue to integrate elements of mobile health to enhance accessibility and engagement for parents. Fifth, researchers should also collect data on participant engagement with interventions to accurately evaluate parents’ interaction with the content. This will help identify the active ingredients and optimal dosages of interventions. Interventions should be designed to be brief and flexible to ensure that parents can receive the full dosage regardless of factors such as hospital length of stay, infant death, returning to work, or feeling overwhelmed by stress. Given the variety of interventions across the studies, there is a clear need for further replication and standardization to develop a consistent approach to mental health support for parents of preterm infants admitted to the NICU.

## 7. Conclusions

Overall, the interventions reviewed in this scoping review illustrate the potential to prevent and treat the psychological impact of the NICU experience on parents. To assist NICU professionals in delivering optimal care to parents, this scoping review highlights the necessity for further research on effective and feasible interventions to alleviate parental distress in this vulnerable population.

## Figures and Tables

**Table 1:** Summary of Study Characteristics (N = 18).

Characteristic	N (%)
Prevention Studies Psychotherapeutic Educational–Behavioral Educational	11 (61.1%) 4 (22.2%) 5 (27.8%) 2 (11.1)
Treatment Studies Psychotherapeutic Complementary/Alternative medicine	7 (38.8%) 2 (11.1%) 5 (27.7%)
Geographical Setting Urban Multiple (Urban and rural) Unclear	15 (83.3%) 1 (5.6%) 2 (11.1%)
Parent Race-Ethnicity American Indian Asian Black Hispanic/Latino (non-white) Native Hawaiian, Pacific Islander Multi-racial White Not reported	6 (0.5%) 51 (3.8%) 315 (23.8%) 190 (14.3%) 1 (0.1%) 5 (0.4%) 705 (53.1%) 53 (4.0%)

**Table 2:** Characteristics and key findings of Included Articles (N = 18).

Author (s), Year	Participants	Design	Group Comparison	Psychological Distress Measures	Intervention Dose and Modality	Outcome (s)
Prevention Studies (*N* = 11)						
[43]	N = 50 40 birthing parents, 10 non-gestational parents	Pretest–posttest pilot	**Experimental** Digital app: My Brigham Baby	Stress—PSS: NICU Anxiety- GAD-7	A digital smartphone app providing educational resources and support to parents of hospitalized infants, including access to medical records, educational materials, and tools for logging parenting activities. Participants were required to use the app at least once per week.	The PSS: NICU total scores exhibited a slight increase post-App rollout; however, this difference was not statistically significant. The GAD-7 mean total scores decreased from 8.6 ± 5.7 pre-App to 7.4 ± 5.6 post-App, but this change was non-significant.
[16]	N = 50 mothers **Age** M = 31.8 (*sd =* 4.9) **Infant gestational age** M = 31.3 weeks (*sd = 2.8*)	RCT	**Experimental** CBT **Control** NICU standard care	Depression—BDI-II Stress—SASRQ Trauma—DTS.	3 CBT sessions lasting 45–55 min over the course of approximately 2 weeks during the infant’s hospital stay.	A trend toward reduced trauma and depressive symptoms in the CBT group, no statistically significant difference.
[17]	N = 178 mothers **Age** M = 30.8 (*sd =* 6.8) **Infant gestational age** M = 28.7 weeks (*sd =* 2.7)	Quasi-experiment	**Experimental** mFICare **Control** Usual FCC	Depression—EPDS Stress—PSS: NICU Trauma—PPQ.	The mFICare cohort received FCC, mhealth app, parent group education classes 2–5 times per week; participation in weekday rounds; peer mentorship.	No clinically significant differences between both interventions on PTSD or depression symptoms. mFICare may be more effective in preventing clinically significant PTSD symptoms than FCC alone for mothers experiencing higher levels of NICU related stress.
[18]	N = 240 mothers **Age** M = 26.6 (*sd = 2.2*) **Infant gestational age** ATVV: 27.0(*2.8*) weeks Kangaroo Care: 27.2 weeks (*sd = 2.9*)	RCT	**Experimental** Auditory–tactile–visual–vestibular (ATVV) and Kangaroo Care (KC) **Control** Attention control, discussed safely caring for preterm infant	Depression—CESD Stress—PSS: NICU Anxiety—STAI Trauma—PPQ.	ATVV Consisted of a 15 min session facilitated by a nurse. Mothers provided auditory, tactile, visual, and vestibular stimulation to their infants, starting with voice and gradually adding moderate stroking and eye contact as the infant became alert. KC Guided by a nurse, mothers held their infants in skin-to-skin contact for at least 15 min.	While neither intervention affected measures of psychological distress, those who engaged in KC had an increase in the rate of decline of distress in the first year. Parenting stress was lower for mothers who engaged in an intervention than those who did not.
[38]	N = 412 258 mothers, 154 fathers **Age** M = 31.5 (*sd =* 5.5) **Infant gestational age** M = 31.3 weeks (*sd =* 2.4)	RCT	**Experimental** Creating Opportunities for Parent Empowerment (COPE) **Control** Attention control, provided standard information regarding hospital services and policies.	Depression—BDI-II Stress—PSS: NICU Anxiety—GAD7, STAI.	Trained nurses delivered a 4-phase educational–behavioral intervention, COPE program. (1) 2–4 days after infants’ admission; (2) 2–4 after first intervention; (3) 1–4 days before discharge; (4) 1 week after infant discharge. Utilized audiotape and materials.	Results indicated that mothers in the COPE program experienced significantly less stress in the NICU and less depression and anxiety at 2 months after infant discharge compared to the control group.
[39]	N = 49 parent pairs 49 mothers, 49 fathers **Age** M = 28.2 (*sd =* 0.06) **Infant gestational age** M = 31.9 weeks (*sd =* 2.1)	Quasi-experiment	**Experimental** COPE **Control** NICU Standard Care	Depression—EPDS Stress—PSS: NICU.	Trained nurses delivered a 4-phase educational–behavioral intervention, COPE program. (1) 2–4 days after infants’ admission; (2) 2–4 after first intervention; (3) 1–4 days before discharge; (4) 1 week after infant discharge. Utilized audiotape and materials.	The results of the study indicated that parents who participated in the COPE program reported significantly lower levels of overall parental stress compared to the comparison group. Specifically, there was a significant difference in the parental role subscale of the Parental Stress Scale: Neonatal (PSS) between the COPE parents and the comparison group. However, there were no significant differences between the two groups in terms of postnatal depression scores or length of hospital stay.
[32]	N = 253 mothers **Age** M = 27.5 (*sd =* 4.7) **Infant gestational age** Mean not reported	RCT	**Experimental** COPE **Control** Attention control, provided standard information on hospital services, discharge, and immunizations.	Anxiety—STAI.	Trained nurses delivered a 4-phase educational–behavioral intervention, COPE program. (1) 2–4 days after infants’ admission; (2) 2–4 after first intervention; (3) 1–4 days before discharge; (4) 1 week after infant discharge. Utilized audiotape and materials.	Results suggests COPE intervention significantly predicted lower levels of (younger than 21) mothers’ anxiety levels at 2–4 days post intervention.
[33]	N = 59 mothers **Age** M = 30.9 (*sd = 5.2*) **Infant gestational age** M = 30 weeks (*sd =* 2.8) Multimedia M = 30 weeks (*sd =* 2.5) Paper	RCT	**Experimental** A multimedia tablet providing education regarding preterm birth and infant care to supplement verbal counseling **Comparison** Handout providing education regarding preterm birth and infant care	Anxiety—STAI.	Handout providing education regarding preterm birth and infant care.	Both the paper handout and multimedia tablet were equally and significantly effective at decreasing state anxiety. Participants were less likely to review all the educational materials on the tablet compared to the paper handout.
[35]	N = 105 mothers **Age** M = 32.4 (*sd =* 5.9) **Infant gestational age** M = 30.9 weeks (*sd =* 3.0)	RCT	**Experimental** Trauma-Focused CBT (TF-CBT) **Comparison** One 45 min information session on NICU policies and parenting preterm infants.	Depression—BDI-II Stress—PSS: NICU, SASRQ Anxiety—BAI Trauma—DTS, TEQ.	Intervention included six sessions of a manualized TF-CBT and lasted 3 to 4 weeks with one or two 45–55 min sessions administered weekly. Sessions facilitated by trained therapists.	The intervention was effective in reducing symptoms of trauma and depression in mothers of preterm infants, with significant improvements in trauma symptoms and depression scores compared to an active comparison group. Although both groups showed a significant decline in anxiety scores without a significant difference between them.
[36]	N = 105 mothers **Age** M = 32.4 (*sd =* 5.9) **Infant gestational age** M = 30.9 weeks (*sd =* 3.0)	RCT	**Experimental** Trauma-Focused CBT (TF-CBT) **Comparison** Usual care? One 45 min information session on NICU policies and parenting preterm infants.	Depression—BDI-II Stress—PSS: NICU, SASRQ Anxiety—BAI Trauma—DTS and TEQ.	Intervention included six sessions of a manualized TF-CBT and lasted 3 to 4 weeks with one or two 45–55 min sessions administered weekly. Sessions facilitated by trained therapists.	Mothers in the TF-CB group had significantly fewer symptoms anxiety (*p* = 0.001), depression (*p* = 0.002), and trauma (*p* =< 0.001), than mothers in the control group at 6 months.
[37]	N = 50 mothers **Age** M = 27.8 (*sd =* 7.0) **Infant gestational age** M = 28.5 weeks (*sd =* 3.5)	RCT	**Experimental** Solving Education (PSE) **Comparison** NICU Standard Care	Depression—QIDS Stress—PSS Trauma—MPSS-SR.	Manualized CBT consisting of 4 weekly individual sessions focused on problem-solving conducted at a location of the mother’s choice (i.e., hospital, home). Sessions facilitated by trained multi-disciplinary graduate students.	No statistically significant decrease in depression symptoms in mothers in the PSE group compared to the control group.
Treatment Studies (n = 7)						
[30]	N = 105 mothers **Age** M = 32.3 (*sd =* 6.0) **Infant gestational age** M = 30.63 weeks (*sd* = 2.9)	RCT	**Experimental** CBT **Control** NICU standard care	Depression—BDI-II Stress—PSS: NICU, SASRQ Anxiety—BAI, Trauma—DTS.	Three 45–55 min CBT sessions over 2 weeks during the infant’s NICU stay.	Mothers in the CBT group showed a trend towards reducing trauma and depressive symptoms but did not show statistically significant results.
[31]	N = 20 mothers **Age** M = 27.3 (*sd =* 6.4) **Infant gestational age** M = 28 weeks (*sd =* 2.3)	Pretest–posttest feasibility	**Experimental** Relaxation guided imagery **Control** No control group	Depression—CES-D Stress—PSS Anxiety- STAI-AD.	Listening to a 20 min RGI recording at least once daily for 8 weeks. Participants were instructed to listen to one track for 2 weeks before they switched to the next track. During the last 2 weeks of study, participants could choose to listen to whichever track they most preferred. Short weekly phone call by an RA for participant to estimate how many times they had listened to the CD.	While the study found that the intervention was feasible and moderately helpful in reducing stress, the specific effects on depression and anxiety were not statistically significant. However, there were positive trends indicating lower perceived stress.
[40]	N = 28 26 mothers, 2 fathers **Age** M = 29.2 (*sd =* 6.8) **Infant gestational age** M = 29.2 weeks (*sd =* 2.6)	Feasibility	**Experimental** Mindfulness-based training session **Control** No control group	Stress—PSS: NICU.	A trained instructor facilitated individual sessions teaching parents mindfulness techniques, with the encouragement to practice these both at their infant’s bedside and away from the hospital.	Parents reported satisfaction with the intervention, however, there was not a significant difference in parental stress levels post-intervention.
[41]	N = 40 39 mothers, 1 father **Age** M = 26.4 (*sd =* 8.1) **Infant gestational age** M = 31.6 weeks (*sd =* 4.0)	Pretest–posttest	**Experimental** Art-based occupation group **Control** No control group	Anxiety—STAI.	Occupational therapist and art therapist facilitated 2-h art-based scrapbooking group sessions. An interdisciplinary team of professionals assisted with group.	Results indicated a significant reduction in state anxiety. Qualitative findings from the study suggested that parents found the scrapbooking activity to be a distraction from their worries, calming, relaxing, and enjoyable. It provided them with a sense of hope for the future beyond the NICU and connected them with other parents in similar situations, reducing their isolation and offering support.
[42]	N = 44 37 mothers, 7 fathers **Age** M = 30.2 (*sd =* 6.6) **Infant gestational age** M = 29.6 weeks (*sd =* 3.0)	RCT—Pilot	**Experimental** Mindfulness-based Neurodevelopmental Care **Control** NICU Standard Care	Stress—PSS: NICU.	Trained research professionals taught a one-on-one educational session for parents of preterm infants in the NICU, teaching mindfulness techniques and structured neurodevelopmental care training within 10 days of enrollment	No significant difference in between-group comparisons of stress, however, parents in the experimental group showed a significant decrease in stress scores from enrollment to discharge.
[44]	N = 97 67 mothers, 30 fathers **Age** M = 27.9 (*sd =* 5.0) **Infant gestational age** M = 35.6 weeks (*sd =* 3.8)	RCT	**Experimental** Journaling **Control** NICU Standard Care	Anxiety—HADS.	Participants journaled for a minimum of 2 weeks and a maximum of 4 weeks. Journal included blank pages as well as 10 prompts to guide parent reflection.	Parents in the intervention group showed a decrease in anxiety, although not statistically significant, while depression remained unchanged. Fathers who participated in the journaling program experienced a significant decrease in anxiety compared to mothers.
[34]	N = 23 mothers **Age** M = 27.9 (*sd =* 6.0) **Infant gestational age** M = 31.6 weeks (*sd =* 5.3)	Pre–posttest design Feasibility study	**Experimental Group** Listening sessions	Depression– EPDS Anxiety—BAI.	Six, 45–60 min listening visits over one-month period.	Significantly reduced depressive and anxiety symptoms, over half showing clinically meaningful improvement and high satisfaction with the intervention.

ATVV = Auditory–tactile–visual–vestibular; BAI = Beck Anxiety Inventory; BDI-II = Beck Depression Inventory—Second Edition; COPE = Creating Opportunities for Parent Empowerment; DTS = Davidson Trauma Scale; EPDS = Edinburgh Postnatal Depression Scale; KC = Kangaroo Care; PSS = Perceived Stress Scale; PSS: NICU = Perceived Stress Scale: Neonatal Intensive Care Unit; SASRQ = Standford Acute Stress Reaction Questionnaire; STAI-AD = State-Trait Anxiety Inventory for Adults.

## Data Availability

The original contributions presented in this study are included in the article. Further inquiries can be directed to the corresponding author.

## Abbreviations

The following abbreviations are used in this manuscript:

NICU	Neonatal Intensive Unit
CBT	Cognitive behavioral therapy
COPE	Creating Opportunities for Parent Empowerment
mFICare	mobile-enhanced Family Integrated Care
